# AimSeg: A machine-learning-aided tool for axon, inner tongue and myelin segmentation

**DOI:** 10.1371/journal.pcbi.1010845

**Published:** 2023-11-17

**Authors:** Pau Carrillo-Barberà, Ana Maria Rondelli, Jose Manuel Morante-Redolat, Bertrand Vernay, Anna Williams, Peter Bankhead

**Affiliations:** 1 Centro de Investigación Biomédica en Red sobre Enfermedades Neurodegenerativas (CIBERNED), Universitat de València, Valencia, Spain; 2 Departamento de Biología Celular, Biología Funcional y Antropología Física, Universitat de València, Valencia, Spain; 3 Instituto de Biotecnología y Biomedicina (BioTecMed), Universitat de València, Valencia, Spain; 4 Centre for Genomic & Experimental Medicine, Institute of Genetics and Cancer, University of Edinburgh, Edinburgh, United Kingdom; 5 Centre for Regenerative Medicine, Institute for Regeneration and Repair, University of Edinburgh, Edinburgh BioQuarter, Edinburgh, United Kingdom; 6 MS Society Edinburgh Centre for MS Research, Edinburgh BioQuarter, Edinburgh, United Kingdom; 7 Centre d’imagerie, Institut de Génétique et de Biologie Moléculaire et Cellulaire CNRS UMR 7104—Inserm U 1258, Illkirch, France; 8 Edinburgh Pathology and CRUK Scotland Centre, Institute of Genetics and Cancer, University of Edinburgh, Edinburgh, United Kingdom; Brown University, UNITED STATES

## Abstract

Electron microscopy (EM) images of axons and their ensheathing myelin from both the central and peripheral nervous system are used for assessing myelin formation, degeneration (demyelination) and regeneration (remyelination). The g-ratio is the gold standard measure of assessing myelin thickness and quality, and traditionally is determined from measurements made manually from EM images–a time-consuming endeavour with limited reproducibility. These measurements have also historically neglected the innermost uncompacted myelin sheath, known as the inner tongue. Nonetheless, the inner tongue has been shown to be important for myelin growth and some studies have reported that certain conditions can elicit its enlargement. Ignoring this fact may bias the standard g-ratio analysis, whereas quantifying the uncompacted myelin has the potential to provide novel insights in the myelin field. In this regard, we have developed AimSeg, a bioimage analysis tool for axon, inner tongue and myelin segmentation. Aided by machine learning classifiers trained on transmission EM (TEM) images of tissue undergoing remyelination, AimSeg can be used either as an automated workflow or as a user-assisted segmentation tool. Validation results on TEM data from both healthy and remyelinating samples show good performance in segmenting all three fibre components, with the assisted segmentation showing the potential for further improvement with minimal user intervention. This results in a considerable reduction in time for analysis compared with manual annotation. AimSeg could also be used to build larger, high quality ground truth datasets to train novel deep learning models. Implemented in Fiji, AimSeg can use machine learning classifiers trained in ilastik. This, combined with a user-friendly interface and the ability to quantify uncompacted myelin, makes AimSeg a unique tool to assess myelin growth.

## Author summary

Myelin is formed by specialised cells that wrap themselves around axons and has a major role in the function, protection, and maintenance of nerves. These functions are disturbed by demyelinating diseases, such as multiple sclerosis. In this work we present AimSeg, a new tool based on artificial intelligence algorithms (machine learning) to assess myelin growth from electron microscopy images. Whereas standard metrics and previous computational methods focus on quantifying compact myelin, AimSeg also quantifies the inner myelin tongue (uncompacted myelin). This structure has been largely overlooked despite the fact that it has an important role in the process of myelin growth (both during development and in the adult brain) and recent studies have reported morphological changes associated with some diseases. We report the performance of AimSeg, both as a fully automated approach and in an assisted segmentation workflow that enables the user to curate the results “on-the-fly” while reducing human intervention to the minimum. Therefore, AimSeg stands as a novel bioimage analysis tool that meets the challenges of assessing myelin growth by supporting both standard metrics for myelin evaluation and the quantification of the uncompacted myelin in different conditions.

## Introduction

**Fig 1:**
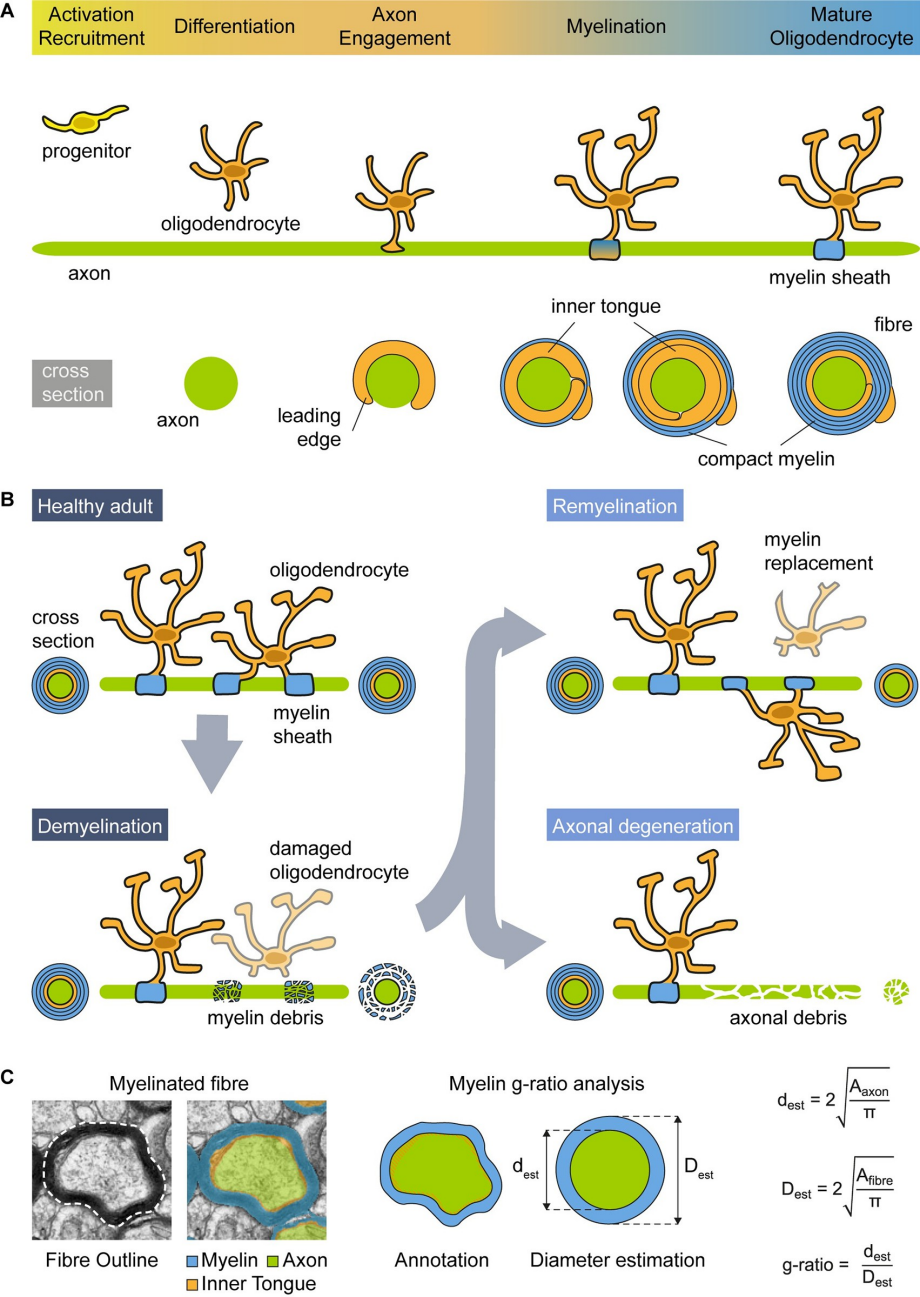
The myelin sheath plays a crucial role in the nervous system, making imaging essential for studying myelin formation, degeneration, and regeneration. **(A)** Schematic representation of the process of myelin formation in the central nervous system. **(B)** Myelin formation is an ongoing process, starting during development (myelination) and continuing throughout lifespan. Myelin regeneration (remyelination) can occur in response to demyelination. Failure in remyelination contributes to axonal degeneration. **(C)** Conventionally for myelin g-ratio analysis, the axon area (green) has been annotated at the inner edge of the compact myelin, thus ignoring the contribution of the area occupied by the inner tongue (diagonal orange stripes). The myelin g-ratio is determined by assimilating the axon and fibre areas to circles to estimate their respective diameters. This conventionally disregards any area contribution from the inner tongue.

The myelin sheath allows faster, saltatory conduction of nerve impulses along the underlying axon without the need to increase axon diameter [1,2]. Moreover, this lipid-rich insulating layer also provides structural protection and metabolic support to the underlying axons [3]. The myelin sheath consists of plasma membrane from either oligodendrocytes (in the central nervous system [CNS]) or Schwann cells (in the peripheral nervous system [PNS]) wrapped around axons, and is discontinuous around their length, separated by nodes of Ranvier [4]. These cells extend cytoplasmic-filled membrane processes that are guided to reach and ensheath the axon. Myelin growth occurs by the wrapping of the leading edge of the myelin membrane process (henceforth the inner tongue) around the axon, progressing underneath the previously deposited membrane in concert with the lateral extension of the individual myelin layers along the axons [5]. Myelin compaction is initiated after a few wraps, occurring first in the outermost myelin layer and progressively spreading inwards, lagging behind the inner tongue to avoid its premature compaction. During developmental myelination, the inner tongue is enlarged but it narrows as myelin matures [4,6]. Once active myelination is completed, a smaller inner tongue remains in adult myelinated fibres [5,7] (see Fig 1A), except as recently discovered in the context of some diseases [8].

Myelination is a process that takes place during development [4]. Nevertheless, in the context of demyelinating conditions like multiple sclerosis (MS), there is potential for damaged myelin sheaths to be replaced through remyelination, a process carried out in the CNS by oligodendrocytes [9]. However, when remyelination does not occur, it becomes impossible to restore energy-efficient conduction, and the supportive function of myelin is forfeited. This results in energy deficiency, disruptions in axonal transport, and ultimately the degeneration of axons [9] (see Fig 1B).

Generally, most myelinated fibres have a ratio of axon to fibre diameters (g-ratio; see Fig 1C) close to the optimal value for conduction velocity of neural electrical impulses, estimated from theoretical models in the PNS and the CNS [10,11]. Additionally, larger diameter axons have more myelin wraps (thicker myelin sheath) and a lower g-ratio [9,12,13]. The g-ratio is widely utilised by the scientific community as a functional and structural index of optimal axonal myelination, and for assessing remyelination following myelin loss. Defects in myelination in the CNS can be assessed in this way in neurodevelopmental disorders [14–17], demyelinating diseases (e.g., MS) [18,19], neurodegenerative diseases [20–22], as well as in rodent models of myelin abnormalities [8,23–26]. Moreover, the remyelination process in MS is characterised by thinner myelin sheaths for the diameter of the axon, giving higher g-ratios [27,28], an extensively utilised trait for discriminating between areas of remyelination and developmental myelination (see Fig 1B).

G-ratios are commonly calculated on electron microscopy (EM) images of chemically fixed samples, though progress has been made to try and measure these *in vivo* on MR brain scans in humans [19,29]. Despite the wide applicability and functional relevance, the g-ratio neglects the inner tongue (see Fig 1C). This is because, for its calculation, the “axon” is usually defined as the inner edge of the compact myelin, which is more readily identifiable by both researchers and computational techniques. Nonetheless, an enlarged inner tongue will bias the standard g-ratio analysis by overestimating the diameter of the axon. Consequently, researchers are adopting alternative ways to perform the g-ratio analysis to assess myelination/remyelination. For example, a “corrected g-ratio” accounting for the enlarged inner tongue has been recently proposed [26]. Moreover, recent studies have reported an enlarged or abnormal inner tongue in transgenic mice (e.g. 2′,3′-cyclic nucleotide 3′-phosphodiesterase (CNP)-deficient mice [23,30], in conditional knock-out of activin co-receptor *Acvr1b* [31] and of *Pten* [5] in oligodendrocytes), and in animal models of autoantibody-mediated- and cuprizone-induced-demyelinating disease [8,25], suggested to be secondary to stressed axons with a compensatory increase in need for metabolic support from the oligodendrocyte via the inner tongue.

Several bioimage analysis approaches have been developed to analyse myelin thickness [32–38]. Many of these approaches are implemented in semi-automated workflows that frequently require several post-processing steps. Deep learning approaches have also been applied [39,40] to segment the individual fibres and their corresponding compacted myelin. Additionally, there are methods available for the analysis of 3D EM images [41]. However, their wide application by researchers has been limited, as still only few are publicly available, well documented, and/or the code made accessible through open-source licensing. Therefore, analysis of myelin thickness from EM images is still largely performed manually by investigators, which is time-consuming and prone to selection bias, thus contributing to limited reproducibility. Notably, all the above-mentioned methods ignore the inner tongue and do not support its quantification.

This has motivated us to develop an open-access tool, named AimSeg, for the segmentation of the axon, the inner tongue, and the compact myelin from EM data. Our goal has been to enable a more thorough assessment of the myelin sheath thickness, while decreasing the need for manual annotation, and saving time. AimSeg uses supervised machine learning (ML) methods implemented in ilastik [42] to improve the segmentation of the fibre components, and combines automated image processing with interactive user-editing stages in Fiji [43]. AimSeg automatically stores all the generated regions of interest (ROIs) in different subsets of axonal components interrelated between them and the results table by the axon IDs. The workflow code for AimSeg–implemented as a Groovy script–is open-source and the pre-trained ilastik classifiers are publicly available together with user documentation.

AimSeg training was conducted on transmission EM (TEM) images of corpus callosum tissue samples obtained from mice that were undergoing remyelination following a unilateral toxin-induced demyelination lesion in the corpus callosum (see Fig 1B). We have tested AimSeg on both i) a validation dataset that includes TEM images of remyelinating mice and ii) a control dataset composed of images from a healthy specimen, obtaining similar results. The corpus callosum was chosen as it is a highly myelinated white matter region within the CNS, commonly affected in CNS diseases and therefore used often in preclinical studies. Furthermore, segmentation of the compacted and uncompacted myelin is more technically challenging after remyelination compared with the relatively straightforward task of segmenting normal myelinated fibres. As a result, our training and validation data takes into account a variety of features, including myelinated and unmyelinated axons, and a wide range of fibres with different myelin and inner tongue thickness, thereby extending the utility of AimSeg for the segmentation of myelinated fibres cross-sections from TEM images.

## Results

### A bioimage analysis workflow for the analysis of myelinated axons

**Fig 2:**
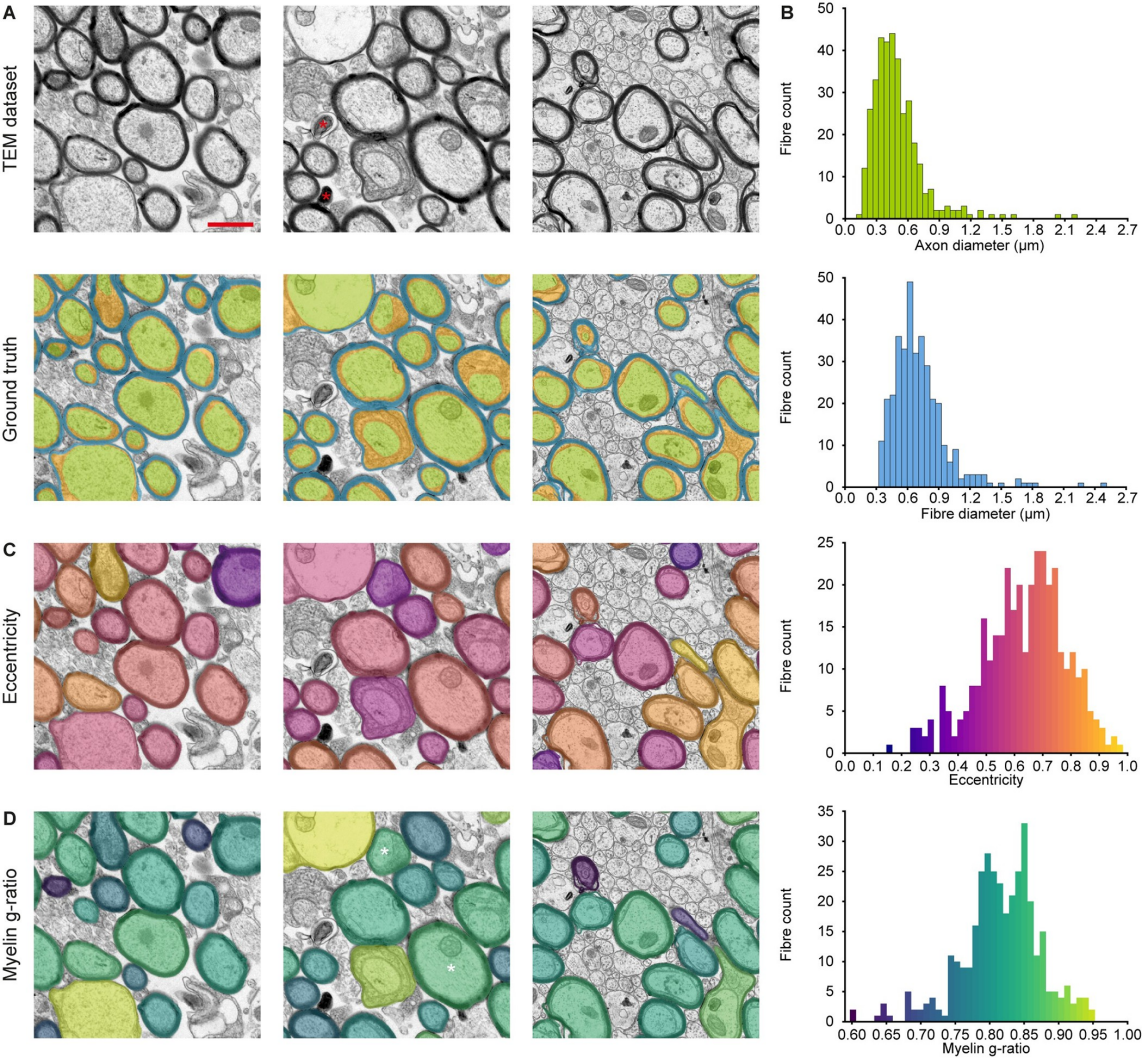
Validation ground truth for the segmentation of fibre cross-sections on electron microscopy images separating the myelin sheath components (compact myelin and inner tongue) from each other and from the axon. **(A, top)** Examples of transmission electron microscopy (TEM) images of the corpus callosum from adult mice undergoing remyelination after inducing a demyelinating lesion. Technical artefacts of no interest, degraded myelin debris and degenerated dark axons (red asterisk) were not included. Scale bar (red line) = 1 μm. **(A, bottom)** Manual segmentation of the compacted myelin (blue), the inner tongue (orange), and the axon (green). **(B-D)** Diversity of axon/fibre size, shape or myelin thickness. **(B)** Histograms representing different metrics determined from the manual annotations. **(C)** The fibres are colour-coded based on the histogram bins to represent the distribution of fibre eccentricity, describing how much a fibre section diverges from a circle, with 0.0 representing a perfect circle. **(D)** The fibres are colour-coded based on the histogram bins that illustrate their myelin g-ratio distribution (ratio of diameter of the area enclosed by the innermost compact myelin border and the diameter of the whole fibre). Higher g-ratios correspond to thinner myelin, with 1.0 representing the complete absence of myelin sheath. It is worth noting that this metric does not account for the presence of the inner tongue. As a result, two fibres, one with a shrunken inner tongue and another with an enlarged inner tongue, can exhibit similar myelin g-ratios (white asterisks).

**Fig 3:**
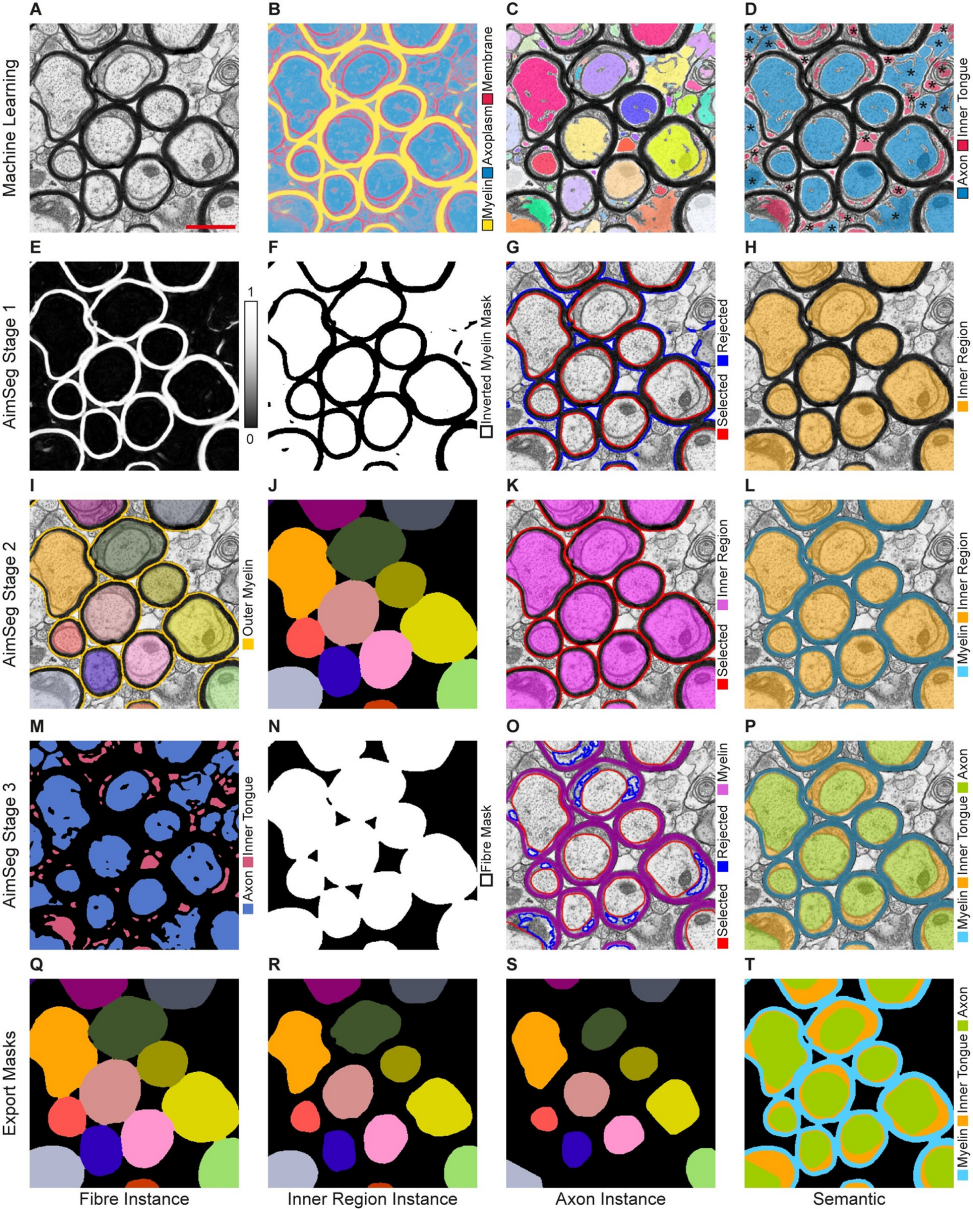
Main steps of the AimSeg bioimage analysis workflow. **(A-D)** AimSeg combines two machine learning (ML) classifiers for pixel and object classification. First, the pixel classifier uses **(A)** the electron microscopy (EM) data to generate a **(B)** probability map. Then, **(C)** potential axon instances are segmented from the axoplasm probabilities. **(D)** The object classifier scores each instance as an axon or inner tongue. Objects outside myelinated fibre cross-sections (marked with an asterisk) will be eliminated in the next steps. **(E-H)** AimSeg Stage 1 uses **(E)** the myelin probability channel to get an **(F)** inverted mask. **(G)** This mask is then analysed to identify the elements within the innermost compact myelin border, which we call the ‘inner region’, and to exclude those representing the background. These identified elements are categorised as either selected or rejected ROIs, respectively. Running the supervised mode (optional), the user can easily toggle the ROI selection group (selected/rejected) or use the ImageJ’s selection tools to add/edit ROIs. **(H)** Semantic segmentation at the end of Stage 1. **(I-L)** AimSeg Stage 2 **(I)** uses the inner region labels as seeds that expand to fill myelin regions generating **(J)** a label mask for the fibres, which is processed to get **(K)** the fibre ROIs. **(L)** Semantic segmentation at the end of Stage 2. **(M-P)** AimSeg Stage 3 combines **(M)** the prediction of axon and inner tongue instances with **(N)** the fibre binary mask. **(O)** This ensures that only myelinated axons are selected. Instances classified as inner tongue are marked as rejected ROIs in the supervised mode. **(P)** Semantic segmentation at stage 3. **(Q-T)** AimSeg combines the gathered sets of ROIs to conduct a thorough analysis of myelinated axons. In this process, AimSeg assigns labels to the instances of **(Q)** the fibre, **(R)** the inner region and **(S)** the axon establishing a hierarchical relationship among instances within the same myelinated axon. **(T)** Additionally, AimSeg generates a semantic mask, where each pixel is categorised as background, axon, inner tongue, or compact myelin. Scale bar (red line) = 1 μm.

In contrast to previous image analysis methods that were developed to calculate conventional g-ratios based upon segmenting fibres and their compact myelin alone [32–40], our goal was to develop a method to separate and analyse each of the fibre components (the axon, the compact myelin and the inner tongue) from TEM images (see Figs 1 and 2). To this end, it is necessary to outline the borders of the axon, the innermost and the outermost compact myelin. AimSeg achieves this through the segmentation of three objects with a hierarchical relationship: the fibre cross-section, the region enclosed by the innermost compact myelin border (henceforth inner region), and the axon. The combination of these masks allows the calculation of both the standard g-ratio and other metrics for the quantification of the inner tongue area. Our strategy relies on supervised ML methods based on random forests, which have been demonstrated to be useful to analyse complex images such as those acquired through EM [44,45]. AimSeg can be applied as a fully automated image processing workflow or enable an assisted segmentation approach that includes interactive user-editing. Our workflow makes use of open-source bioimage analysis software (ilastik [42] and Fiji [43]). The AimSeg core pipeline is a Fiji script that takes as an input a series of files previously generated using ML classifiers trained using ilastik (see Fig 3).

The workflow starts with two classifiers (pixel and object classification; see Fig 3A–3D) trained using supervised ML methods implemented within ilastik–although AimSeg has the potential to use classifiers trained by means of any other ML toolkit. For this work, we trained our classifiers on TEM images acquired on remyelinating tissue; specifically, the dataset includes five images of the corpus callosum from four adult mice after a demyelinating lesion was induced. Users can directly apply the ready-to-use classifiers pre-trained for this work, improve them by adding their own raw data and annotations, or train new classifiers from scratch by following the guidelines provided within the AimSeg documentation.

The AimSeg pipeline is divided in three sequential stages aimed to segment each of the three fibre components from each of the fibre cross-sections in the image. In Stage 1, the myelin probability map is processed to segment the inner region (see Fig 3E–3H), which is used as a seed to get the fibres in Stage 2 (see Fig 3I–3L). Finally, axons are segmented from the object classifier predictions in Stage 3 (see Fig 3M–3P). AimSeg includes optional post-processing that can be applied to automatically correct the axon segmentation using different methods.

Additionally, after the automated steps, each stage includes an optional user-editing step that allows manual amendment of any segmentation inaccuracies before proceeding to the next stage. At these points, the user can delete ROIs, use the Fiji selection tools to edit or add new ROIs or use a series of shortcuts provided in AimSeg to interact with the ROIs while reducing the user intervention. Additionally, those ROIs filtered out during the automated processing are also shown as rejected-mode ROIs at Stage 1 and 3, so the user can toggle them to the selected-mode without drawing them from scratch (see Fig 3G and 3O).

Once the three ROI sets have been generated, an automated pipeline within the Fiji-implemented workflow is set to both post-process the three ROI sets and to extract the quantitative features. The post-processing step aims to: i) remove any residual pixels that may have been left by the user–during the manual editing step–by keeping only the biggest region on composite ROIs (stages 1 and 2; axons are allowed to be composite ROIs); ii) ensure that, as expected, the ROIs of the innermost elements do not overflow into the outer ones (e.g., the axon ROI should never break through the inner region ROI; stages 1 and 3); iii) optionally duplicate the inner region ROI in case an axon ROI has not been selected for a fibre due to a shrunken inner tongue; and iv) constructing a hierarchical relationship between the three ROI sets corresponding to different fibre components (see Fig 3Q–3S). The latter step is important for the performance of a meaningful quantification, and enables the user to trace results from the final measurements table back to the image dataset. Finally, the area of each ROI in the hierarchy is extracted and summarised in a results table.

AimSeg includes additional commands to visualise the final semantic segmentation as an overlay on top of the original image (see Fig 3P), and to export the definitive ROI sets as instance (see Fig 3Q–3S) and semantic (see Fig 3T) segmentation masks.

### Assessment of the automated and supervised segmentation

First we evaluated the learning efficiency of the ML methods used to generate the pixel and object classifiers. The results obtained comparing ilastik classifiers trained on different numbers of samples (1–5 images) showed a limited effect on the final AimSeg output. Small and sparsely annotated datasets generated results close to classifiers trained with more data. This suggests that the classifiers reach their maximum precision with few annotations; further training does not seem to improve the results, and may even be counterproductive (S1 Fig).

**Fig 4:**
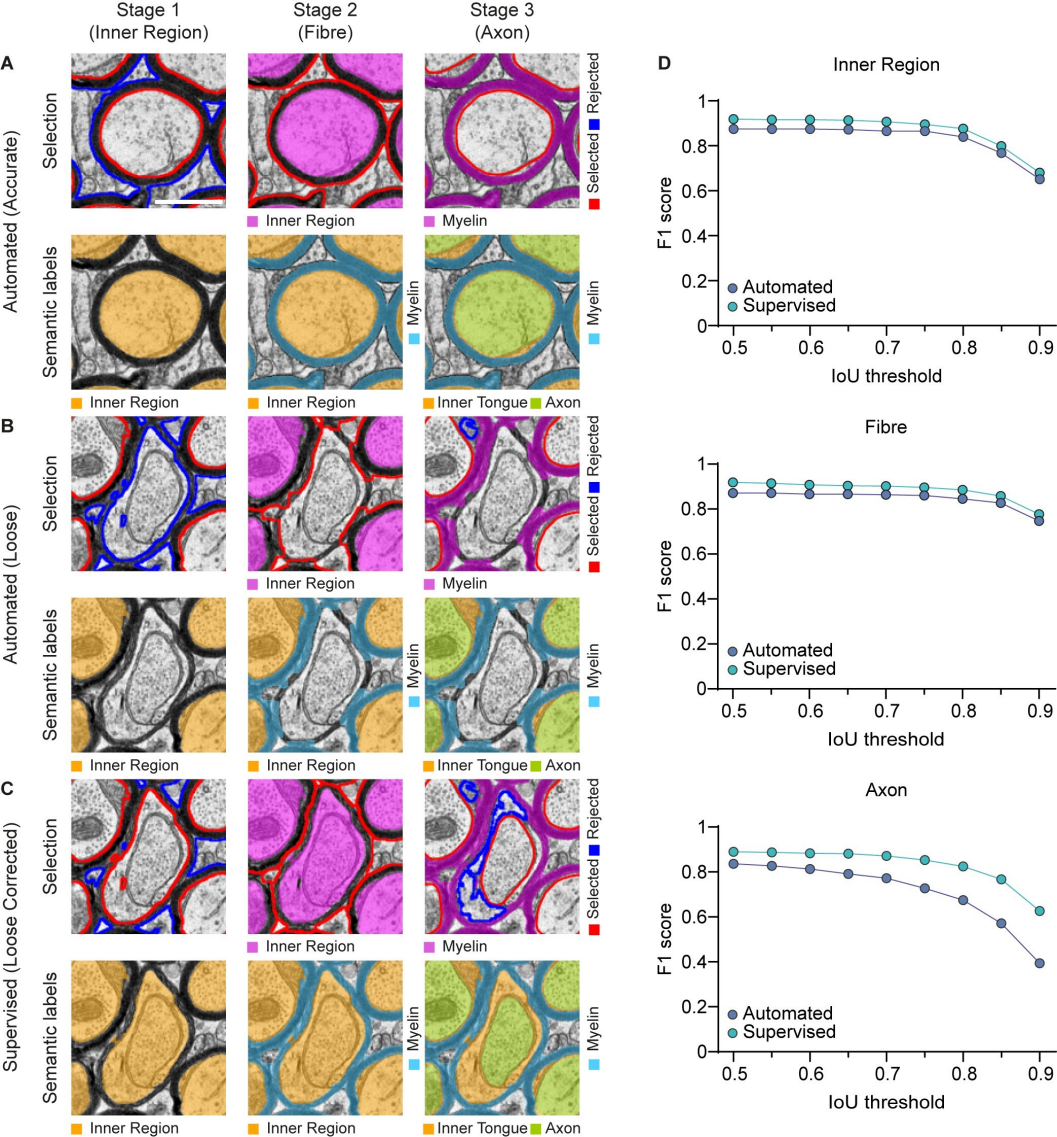
AimSeg segmentation performance, assessed independently for the three detections performed sequentially by AimSeg: the inner region (i.e., the axon plus the inner tongue), the fibre and the axon. Evaluation of the instance segmentation performed either in **(A, B)** automated or **(C)** supervised modes. At first, since no user intervention was allowed, results included both **(A)** accurate and **(B)** loose segmentations. Note that skipping an inner region at Stage 1 caused the myelin mask of the surrounding fibres to overflow at Stage 2. **(C)** The supervised mode allows the user to curate the AimSeg selection during the user-edited stages. Note that toggling the rejected inner region at Stage 1 solves the overflowing issue at Stage 2 and facilitates the automated detection of the corresponding fibre and axon. **(D)** Quantitation of the segmentation performance is based on the F1 score, an object-based metric, plotted for increasing intersection over union (IoU) thresholds for estimating the shape matching accuracy in both the automated and the human-supervised results. Scale bar (white line) = 0.5 μm.

To quantitatively assess the performance of our strategy, we compared the segmentation output obtained using our workflow to analyse the images of the validation ground truth (five images from four mice), manually annotated by an expert (see Fig 2). As discussed above, one important aspect of AimSeg’s design is the user-supervised step that makes it possible to correct or edit the automatic segmentation. Therefore, to evaluate the extent to which such manual steps are necessary or beneficial, we compared the expert’s ground truth with two different workflow outputs: a fully automated workflow without any user intervention (see Fig 4A and 4B), and a supervised (assisted segmentation) workflow that included a limited version of all the user-editing steps. For the evaluation of the supervised mode, the user was allowed to edit the ROI sets by including or discarding ROIs automatically suggested by our tool, but not manually drawing them (see Fig 4C). This allowed us to assess the potential of AimSeg to facilitate the user-assisted segmentation.

We used the “F1 score” to assess the performance of AimSeg during the automated and supervised modes. The average F1 score of all the annotated images was calculated across a range of intersections over union (IoU) thresholds, from 0.5 to 0.9 (in increments of 0.05). The representation of the F1 score along an IoU threshold range allows one to simultaneously look at the correctly identified objects and the pixel-wise closeness of their corresponding ROIs. A higher F1 score denotes a good detection of the object while a lower score corresponds to a poor object detection.

F1 scores were independently computed for the fibre, the inner region, and the axon (see Fig 4D; precision and recall in S2 Fig). The automated approach demonstrated a considerable capability to predict the fibre constituents, with the F1 score being consistently high across the IoU thresholds. We also demonstrate that these results can be substantially improved by allowing the user to review and amend the segmented objects for all three components, even when not allowed to use the selection tools in Fiji to upgrade the ROI selection or to create new ones. AimSeg shortcuts lead to an improved performance at the three stages, being especially relevant in the segmentation of axons. Comparing the average F1 score obtained using either the automated or the supervised AimSeg analysis, we observed that the score increased from 0.83 to 0.87 for the inner region, from 0.85 to 0.88 for the fibre, and from 0.71 to 0.83 for the axon.

### Quantitative validation of myelin analysis

**Fig 5:**
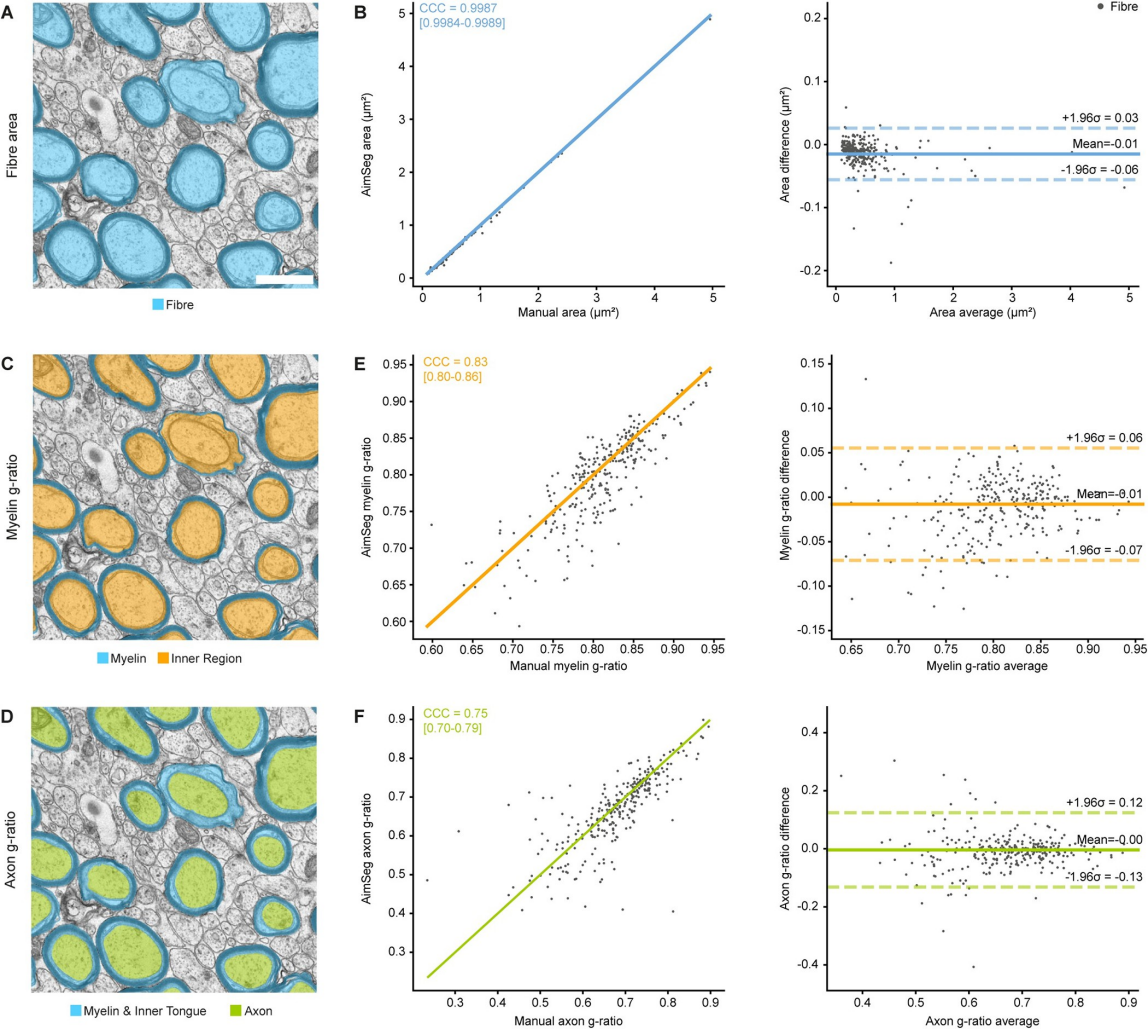
Agreement between the validation ground truth and the AimSeg measurements for the analysis of myelin properties. **(A)** Example image for the semantic segmentation of the fibres. Scale bar (white line) = 1 μm. **(B)** Comparison of the fibre areas obtained by manually segmenting the images or using AimSeg. **(B, left)** The measurement agreement is calculated as the Lin’s concordance correlation coefficient (CCC), **(B, right)** while the measurement bias is assessed by means of a Bland-Altman analysis. Diagonal line in the CCC plot represents perfect agreement (y = x). **(C)** Illustration of the myelin g-ratio measurement, calculated as the ratio of the inner region diameter to the fibre diameter. **(D)** Illustration of the axon g-ratio measurement, calculated as the ratio of the axon diameter to the fibre diameter. CCC and Bland-Altman plot for **(E)** the myelin and **(F)** the axon g-ratios.

After assessing the capability of AimSeg to segment the different elements of the fibre, we wanted to investigate its adequacy to generate accurate measurements for the analysis of the myelin properties. AimSeg enables the extraction of standard fibre features such as axon, inner tongue or myelin areas, which can be combined to calculate different myelin properties. In order to evaluate the agreement between the measurements obtained from the manual annotation and the segmentation obtained with AimSeg, the computed fibre areas (see Fig 5A) were compared using the Lin’s concordance correlation coefficient (CCC). Since the CCC requires a list of matched samples, fibres with an IoU lower than 0.5 were rejected from the analysis (15% false negatives, 5.8% false positives). Fibre areas agreed with a CCC of 0.9987 (95% confidence interval (CI) 0.9984–0.9989). Fibre areas detected using AimSeg were, on average, 0.01 μm^2^ smaller than those manually annotated and the limits of agreement were between -0.06 and 0.03 μm^2^ (see Fig 5B).

Following the segmentation of the inner tongue, we distinguish between two types of “g-ratio”: 1) the classical g-ratio, here called the “myelin g-ratio”, the ratio of inner region to the fibre diameter, describing the thickness of the compact myelin relative to the axon and the inner tongue (see Fig 5C) and 2) the “axon g-ratio”, the ratio of the axonal diameter (excluding the inner tongue) to the fibre diameter, describing the thickness of both the compact and the non compact myelin relative to the axon diameter (see Fig 5D). Therefore, the difference of the myelin and the axon g-ratios can be used as a relative measurement to estimate the inner tongue enlargement, where 0 is equivalent to an absent structure.

Myelin g-ratios agreed with a CCC of 0.83 (95% CI 0.8–0.86). On average, AimSeg analysis returned myelin g-ratios 0.01 smaller than manual segmentation, while the limits of agreement were between -0.07 and 0.06 (see Fig 5E). The CCC for the axon g-ratio was 0.75 (95% CI 0.7–0.79). The mean difference of the axon g-ratios calculated by AimSeg and the validation ground truth is close to zero, even if the limits of agreement were between -0.13 and 0.12 (see Fig 5F).

We aimed to determine if the enhanced segmentation quality achieved through the utilisation of AimSeg shortcuts results in a more precise analysis of myelin properties. As expected, we observed a decrease in the number of fibres rejected by the 0.5 IoU filter when employing the supervised mode (14% false negatives, 0.56% false positives). The CCC of the fibre area remained close to 1, while the myelin g-ratio experienced a slight increase to 0.84 CCC. The axon g-ratio benefited most from the supervised workflow, reaching a CCC of 0.87. The limits of agreement for all the measurements investigated remained largely unchanged (see S3 Fig).

### Performance on non-remyelinating samples

**Fig 6:**
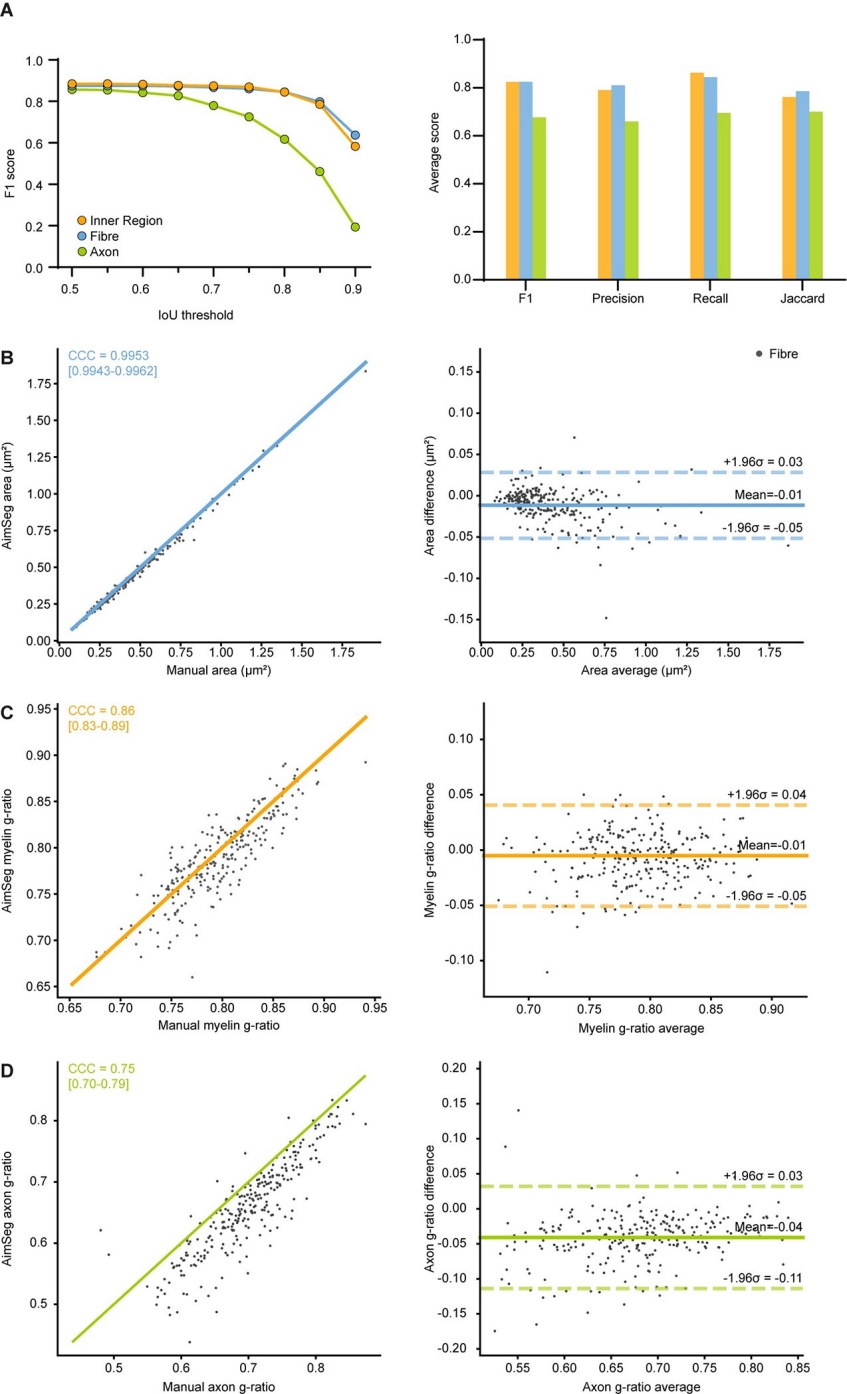
Segmentation metrics and agreement between the control ground truth and the AimSeg measurements for the analysis of myelin properties. **(A)** Segmentation performance for the instances detected at each AimSeg stage. **(A, left)** F1 score plotted for increasing intersection over union (IoU) thresholds. **(A, right)** Average F1 score, precision, recall, and Jaccard index. **(B)** Comparison of the fibre areas obtained by manually segmenting the images or using AimSeg. **(B, left)** The measurement agreement is calculated as the Lin’s concordance correlation coefficient (CCC), **(B, right)** while the measurement bias is assessed by means of a Bland-Altman analysis. Diagonal line in the CCC plot represents perfect agreement (y = x). CCC and Bland-Altman plot for **(C)** the myelin and **(D)** the axon g-ratios.

Tissue undergoing remyelination contains axons whose myelin sheath may present a wide variety of states and, additionally, a high proportion of unmyelinated axons. Therefore, it seemed suitable data for training and validating AimSeg. We also tested its performance on control, healthy, more uniform tissue (without a demyelinating lesion) (see S4 Fig). Overall, the segmentation metrics and the biological baseline proved to be very similar to the validation dataset (see Fig 6A). The statistical analysis performed on the identified objects within the control dataset (with a 5.9% false negative rate and a 6.6% false positive rate) revealed a CCC of 0.9953 (95% CI 0.9943–0.9962) for fibre areas. Fibre areas detected using AimSeg were, on average, 0.01 μm^2^ smaller than those manually annotated and the limits of agreement were between -0.05 and 0.03 μm^2^ (see Fig 6B). Myelin g-ratios on control samples agreed with a CCC of 0.86 (95% CI 0.83–0.89). Myelin g-ratios, on average, were 0.01 smaller than manual segmentation and the limits of agreement were between -0.05 and 0.04 (see Fig 6C). The CCC for the axon g-ratio of the healthy mouse was 0.75 (95% CI 0.7–0.79). On average, the axon g-ratios calculated by AimSeg were 0.04 smaller than those manually annotated. In this case, the limits of agreement were between -0.11 and 0.03 (see Fig 6D).

### Computation time

The time required to manually annotate the validation ground truth per image was approximately one hour compared to an average of 6.34 seconds with automatic processing with AimSeg. We further assessed the computational time required for each automated step of the AimSeg core workflow. The time required for the automated processing steps (i.e., excluding parameterisation, data import and user supervision) of each stage per image was: 0.74 s (Stage 1), 1.97 s (Stage 2), and 2.01 s (Stage 3) and 1.62 s for post-processing and quantification. Therefore, the computational time for automated processing across the three stages was negligible when compared with the highly time-consuming endeavour of annotating the images manually.

## Discussion

The g-ratio is the gold standard for the assessment of the optimal myelination of axons. However, the calculation of this highly used metric neglects the existence of the uncompacted myelin of the inner tongue; a fibre component with relevance during myelination and remyelination, and whose thickness variation may contribute to the identification of both physiological and pathological processes. In fact, our dataset on remyelinating white matter clearly shows that fibres of similar cross-section diameter, but differing in the presence of inner tongue would often render almost identical classic myelin g-ratio values evidencing the risk of overlooking relevant biological conditions. The lack of bioimage analysis tools accounting for the inner tongue makes its quantification a tedious task, requiring the manual annotation of EM images by experts: this is a common bottleneck that often hinders the quantification of larger datasets. Here we present AimSeg, a bioimage analysis tool for axon, inner tongue, and myelin segmentation of fibre-cross sections from EM images.

The AimSeg workflow was built using open-source bioimage analysis software to combine supervised ML with an image processing pipeline to facilitate the annotation of the fibre compartments. To this end, it takes advantage of the user-friendly and interactive ML tools provided by ilastik (which are readily accessible for users without coding experience), the versatility of Fiji, and the interoperability of both toolkits. A post-processing pipeline corrects some common annotation errors and establishes a hierarchical relationship between different ROI sets before quantifying key myelin metrics. In this context, we propose differentiating between the myelin g-ratio, which corresponds to the classic g-ratio, and the axon g-ratio, which takes into account the inner tongue area in addition to the myelin sheath. By combining both metrics, we can identify enlarged inner tongues that may bias myelin analysis, and explore variations in the inner tongue as an independent subject of investigation.

Functioning as a fully automated tool, AimSeg has demonstrated both significant segmentation accuracy and the capacity to produce its measurements for analysing myelin properties that closely align with those obtained directly from the ground truth. However, we have observed occasional underestimation of the axon area likely due to the presence of electron-dense bodies such as mitochondria or neurofilaments within the axoplasm. Additionally, we have noted variations in segmentation performance between well-preserved fibres and those affected by tissue-processing artefacts, emphasising the significance of sample preparation, a common challenge in bioimage analysis. It is important to clarify that AimSeg is designed to segment all myelinated axons independently of their quality. To address these or any other potential segmentation issues, AimSeg has been designed as a flexible tool that includes several selectable automatic operations to correct the predicted axon ROIs, such as automated convex hull estimation. On the other hand, throughout the three segmentation stages, users can make use of an interactive, shortcut-assisted, supervised ROI edition workflow. Our validation results demonstrate that the application of these correction/edition tools clearly improves the segmentation output and the accuracy of the obtained data with minimum impact on the processing time when compared to fully manual annotation.

The end goal of AimSeg is to provide tools that enable more sophisticated bioimage analysis in the field of myelin biology. We have demonstrated AimSeg in combination with conventional ML using random forests, because this provides a flexible workflow that can be readily integrated into different laboratories with minimal effort (i.e. sparsely annotating only a few images is sufficient to train models adapted to a new image type). Although beyond the scope of the current work, this flexibility may be useful for the analysis of images obtained using other microscopy modalities, either EM (scanning electron microscopy, scanning transmission electron microscopy) or optical microscopy (e.g., brightfield, confocal). While the limitation on the spatial resolution achieved by some of these modalities may prevent the analysis of the inner tongue, AimSeg is capable of determining classic myelin g-ratios, for which the higher resolution of TEM is not essential. Moreover, the AimSeg pipeline can readily be adapted to use other pixel and object classifier outputs, for example generated using standard supervised deep learning-based approaches.

Future work will explore training more generalised deep learning models for fibre compartment segmentation and classification, based on training data gathered from different laboratories. This has the potential to make deployment even easier, by removing the need for classifier training on a per-lab, per-modality basis. The efficient, computer-assisted annotation features currently within AimSeg will help in generating the ground truth for such models at scale, while the full AimSeg pipeline will remain important to translate the deep learning outputs into biologically meaningful quantitative results. Any future work, whether conducted by us or the scientific community, can benefit from AimSeg’s ground truth data along with the QuPath scripts for the generation of compatible datasets, all openly shared. Collectively, these complementary assets hold the potential to serve as a foundational resource for projects aiming to achieve a comprehensive segmentation of myelinated axons.

With this work, we contribute to filling the gap between myelin biology and bioimage analysis. We believe that AimSeg may facilitate the study of the long-neglected inner tongue by providing a user-friendly, open-source platform for its quantification. Moreover, our assisted segmentation approach enhances the throughput capability of the analysis while enabling manual annotation. Overall, AimSeg’s features and novel metrics have the potential to support more sensitive and high-throughput approaches to analyse myelin ultrastructure beyond the standard g-ratio.

## Methods

### TEM dataset

Experimental protocols involving mice were performed under UK Home Office project licence PADF15B79 (A.W.) issued under the Animals (Scientific Procedures) Act. Adult mouse corpus callosum tissue from remyelinating and healthy specimens was obtained and processed for EM as described in [18,46]. TEM images used in this study were collected on a JEOL JEM-1400 Plus TEM with GATAN OneView camera at 7.1 K magnification with image dimensions 8.62 μm x 8.62 μm (4096 x 4096 pixels; pixel size 2.1046 x 10^−3^ x 2.1046 x 10^−3^ μm).

### Data preprocessing

AimSeg includes a preprocessing command that can be used to resize and/or normalise the image dataset. If the normalisation option is checked, images are converted to 32-bit and their pixel values are normalised to be floats in a 0 to 1 range. Data normalisation is conducted using ImageJ’s ContrastEnhancer class. Bit conversion uses ImageJ’s ImageConverter class.

The TEM dataset used to train the ilastik classifiers and validate AimSeg was normalised enabling a 1% of saturated pixels and resized using a downsampling factor of 4, generating new data with image dimensions 8.62 μm x 8.62 μm (1024 x 1024 pixels; pixel size 8.4182 x 10^−3^ x 8.4182 x 10^−3^ μm).

### Validation and control ground truth

Evaluation was performed on corpus callosum tissue samples obtained from independent mice undergoing remyelination different from those selected for training the classifiers. Additional tests were conducted on control samples to assess AimSeg performance on samples that had not undergone demyelination. Manual annotations of the three ROIs (axon, inner region, and fibre) were done by a single expert in QuPath [47], thus generating the ground truth for the respective ROI set of entire images. The validation ground truth consists of five TEM images from four mice. The control ground truth consists of three TEM images from a healthy specimen. Annotations were exported using QuPath’s scripting language to generate three independent instance masks (axon, inner region, and fibre images) and one semantic mask (axon, inner tongue, and compact myelin pixels) per image applying a downsampling factor of 4.

### Pixel and object classification training and learning efficiency

ML tools implemented within ilastik 1.3.3post3 [42] were used to perform a pixel classification and an object classification.

Pixel classification was conducted as an ilastik autocontext workflow [42], which performs two sequential pixel classifications using the predictions of the first classifier as additional channels for the input of the second classifier. Four different classes were defined for the first pixel classification: i) compacted myelin, ii) axoplasm iii) membrane–such as the axolemma or the inner tongue membrane–and, iv) mitochondria. The second pixel classifier uses the same classes but merges mitochondria within the axoplasm class to prevent holes on the final axon instances. The pixel classifier uses all the intensity, edge and texture features implemented within ilastik with different σ (0.3, 0.7, 1.0, 1.6, 3.5, 5.0, 10.0, 15.0, 30.0, 50.0).

The object classification pipeline starts performing an instance segmentation, taking as input the probability map generated by the pixel classifier. The axoplasm probability channel is smoothed (σ = 2.0 sigma), thresholded (0.6 threshold) and size-filtered (with objects smaller than 10 pixels rejected) to compute potential axon instances. However, there are electron-lucent structures that can be segmented along the axons, such as cells or inner tongue sections. Therefore, we defined three different classes: two representing axon cross-sections (larger or smaller), and the other representing inner tongue cross-sections. The rest of the objects obtained through the instance segmentation, including cells or unmyelinated axons, are not annotated during the training process, and thus their predictions are ignored. The classifier uses all the shape and intensity distribution features implemented within ilastik for the object classification; conversely, the location features were ignored.

Both pixel and object classifiers were trained interactively using a subset of five images randomly selected as the training set from one mouse. To assess the learning efficiency, five pairs of pixel-object classifiers were trained using a different number of images (from 1 to 5), with an increment of 1 image per step.

### Image processing methods implemented in AimSeg

AimSeg is implemented as a Fiji workflow and maintained through an update site. AimSeg handles the segmentation output as three different types of data, including binary masks, ROIs and label masks. Basic binary operations (erode, dilate, open, close, fill) are implemented as modifications of ImageJ 1.x source code [48]. Logical operators use ImageJ’s ImageCalculator class. Binary reconstruction is part of the morphological operations provided at Fiji’s Morphology update site [49]. Connected components from binary masks are detected, filtered and converted into ROIs with the ImageJ ParticleAnalyzer plugin. AimSeg handles different ROI sets by means of independent ImageJ RoiManager instances. The RoiManager is also used to transform ROIs into binary or label masks. Operations with individual ROIs use ImageJ Roi, PolygonRoi and ShapeRoi classes. These include calculating the convex hull, filling of ShapeRois, filtering PolygonRois contained in ShapeRois by specified criteria, and calculating the intersection of two ROIs. Additionally, ROI erosion and dilation is performed with ImageJ RoiEnlarger plugin. Operations with label masks are implemented using the MorphoLibJ library [50] by accessing the MarkerControlledWatershedTransform2D and ReplaceLabelValues classes. Label masks are transformed into ROIs by means of the ImageJ’s ThresholdToSelection plugin.

### Metrics for the assessment of instance segmentation

To evaluate the performance of the segmentation we used precision, recall and the F1 score. We computed object-based metrics rather than pixel-based metrics because the goal of our pipeline is to perform the instance segmentation of each individual fibre and its components to extract morphometric features. Briefly, the metrics used are based on computing the overlapping degree between the target (T), i.e., the validation ground truth annotated by an expert, and the prediction (P) masks, automatically generated by AimSeg. First, the overlap between T and P is calculated for each object as the intersection over union (IoU) metric (also known as Jaccard index).


$$IoU(T,P)=\frac{T\cap P}{T\cup P}$$ (1)

Where the intersection (T⋂P) is the count of the pixels shared by both T and P, whereas the union (T⋃P) is the count of the pixels that are part of either T, P or both. Therefore, the IoU has a value of 1.0 for identical objects, while a value of 0 indicates that there is no overlap between T and P.

Then, an IoU threshold is set to label each object as a true positive (TP), a false negative (FN) or a false positive (FP).

Precision is determined by the proportion of predicted objects that had a match with the annotated ground truth and is defined as:

$$Precision=\frac{TP}{TP+FP}$$ (2)

Recall is determined by the proportion of target objects that had a match on the prediction mask, calculated as:

$$Recall=\frac{TP}{TP+FN}$$ (3)

The F1 score is defined as the harmonic mean of precision and recall:

$$F1=2\times \frac{precision\times recall}{precision+recall}$$ (4)

Therefore, the F1 score can be calculated as:

$$F1=\frac{2TP}{2TP+FN+FP}$$ (5)

We computed the three metrics along a range of IoU values, since the selection of a single IoU threshold may be considered an arbitrary measure. We excluded IoU values below 0.5 to avoid the conflict of pairing a T object with two P objects, or vice versa. Notably, a perfect overlap is practically unattainable, even when comparing the annotations of two human operators. Therefore, we used a range from 0.5 to 0.9 with increments of 0.05. The average precision, recall and F1 score is calculated as the mean of all the scores obtained at all the IoU thresholds previously described. Additionally, the Jaccard index average is calculated as the IoU mean of all T individual objects.

The assessment of AimSeg segmentation performance (IoU, precision, recall, and F1 score) has been computed using a customised script based on the evaluation pipeline implemented by Caicedo et al [51].

### Correlation and bias analysis for AimSeg myelin quantification

The CCC was used to determine the level of agreement in measurements between myelin metrics obtained from AimSeg segmentation masks and the manual segmentation performed by an expert (validation and control ground truth). Unlike other methods, CCC relies on concordance, not just linearity. Therefore, all the spots in the scatter plot comparing two samples with a perfect CCC are expected to fall on the x = y line. The expected bias was computed by means of a Bland-Altman analysis. The validation of myelin analysis was conducted using a customised script based on the validation pipeline implemented by Matthews et al [52]. Image processing and myelin analysis (fibre area, myelin g-ratio, and axon g-ratio) for correlation and bias analysis were carried out using Python’s skimage [53], numpy [54] and pandas [55] libraries. Since it is not possible to extract reliable myelin metrics from incomplete fibres, CCC and Bland-Altman analyses were performed after eliminating the image borders from both the validation ground truth and the AimSeg prediction.

### Hardware

Computation time quantification was performed on a HP OMEN 15-DC0000NS laptop with an Intel Core i7-8750H processor, 16 GB of RAM and an NVIDIA GeForce GTX 1060 graphic card.

## Supporting information

S1 Fig Learning efficiency of the classifiers. Evaluation of the number of training images required to train efficient classifiers for AimSeg. Different classifiers were used to run AimSeg and compute the F1 score obtained for the segmentation of **(A)** the inner region (axon plus inner tongue), **(B)** the fibre and **(C)** the axon.
(TIF)

S2 Fig AimSeg segmentation precision and recall, assessed independently for the three exported ROI sets: the inner region (i.e., the axon plus the inner tongue), the fibre and the axon. AimSeg was run in automated and supervised modes to compute precision and recall for the segmentation of **(A)** the inner region, **(B)** the fibre, and **(C)** the axon.
(TIF)

S3 Fig Agreement between the control ground truth and the supervised AimSeg measurements for the analysis of myelin properties. **(A)** Comparison of the fibre areas obtained by manually segmenting the images or using AimSeg. **(A, left)** The measurement agreement is calculated as the Lin’s concordance correlation coefficient (CCC), **(A, right)** while the measurement bias is assessed by means of a Bland-Altman analysis. Diagonal line in the CCC plot represents perfect agreement (y = x). CCC and Bland-Altman plot for **(B)** the myelin and **(C)** the axon g-ratios.
(TIF)

S4 Fig Control ground truth for the instance segmentation of fibres and the semantic segmentation of myelin, inner tongue, and axon. **(A, top)** Examples of transmission electron microscopy (TEM) images of the corpus callosum from a healthy, adult mouse. Scale bar (red line) = 1 μm. **(A, bottom)** Manual segmentation of the compacted myelin (blue), the inner tongue, (orange) and the axon (green). **(B-D)** Diversity of axon/fibre size, shape or myelin thickness. **(B)** Histograms representing different metrics determined from the manual annotations. **(C)** The fibres are colour-coded based on the histogram bins to represent the distribution of fibre eccentricity, describing how much a fibre section diverges from a circle, with 0.0 representing a perfect circle. **(D)** The fibres are colour-coded based on the histogram bins that illustrate their g-ratio distribution (ratio of diameter of the area enclosed by the innermost compact myelin border and the diameter of the whole fibre). Higher g-ratios correspond to thinner myelin, with 1.0 representing the complete absence of myelin sheath.
(TIF)
