# Supplementary figures and images for: AimSeg: A machine-learning-aided tool for axon, inner tongue and myelin segmentation
Source: PLoS Comput Biol. 2023 Nov 17;19(11):e1010845. doi: 10.1371/journal.pcbi.1010845 (PMC10691719; doi:10.1371/journal.pcbi.1010845)

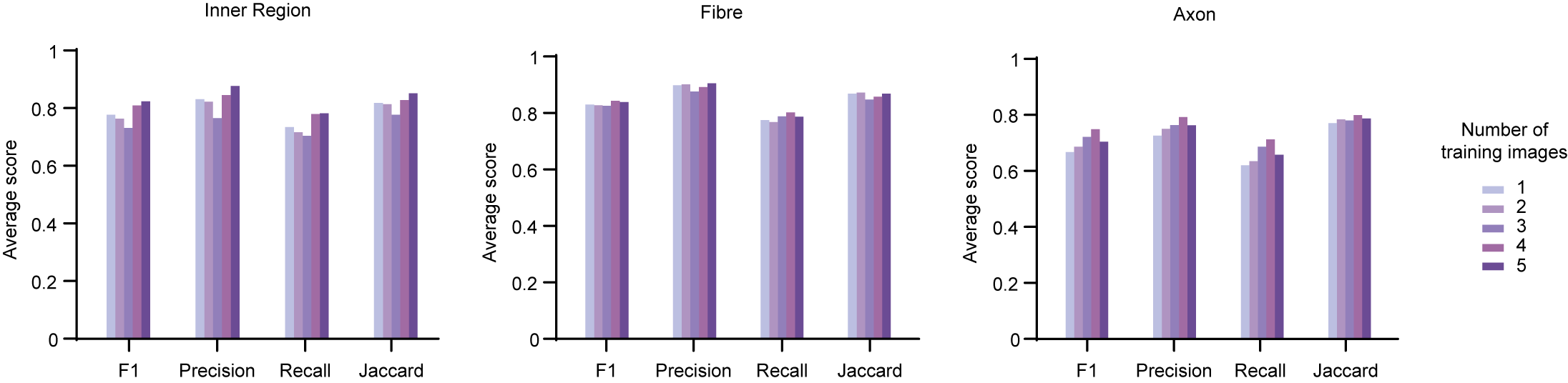

Supplement: S1 Fig — Evaluation of the number of training images required to train efficient classifiers for AimSeg. Different classifiers were used to run AimSeg and compute the F1 score obtained for the segmentation of (A) the inner region (axon plus inner tongue), (B) the fibre and (C) the axon. (TIF) [file pcbi.1010845.s001.tif]

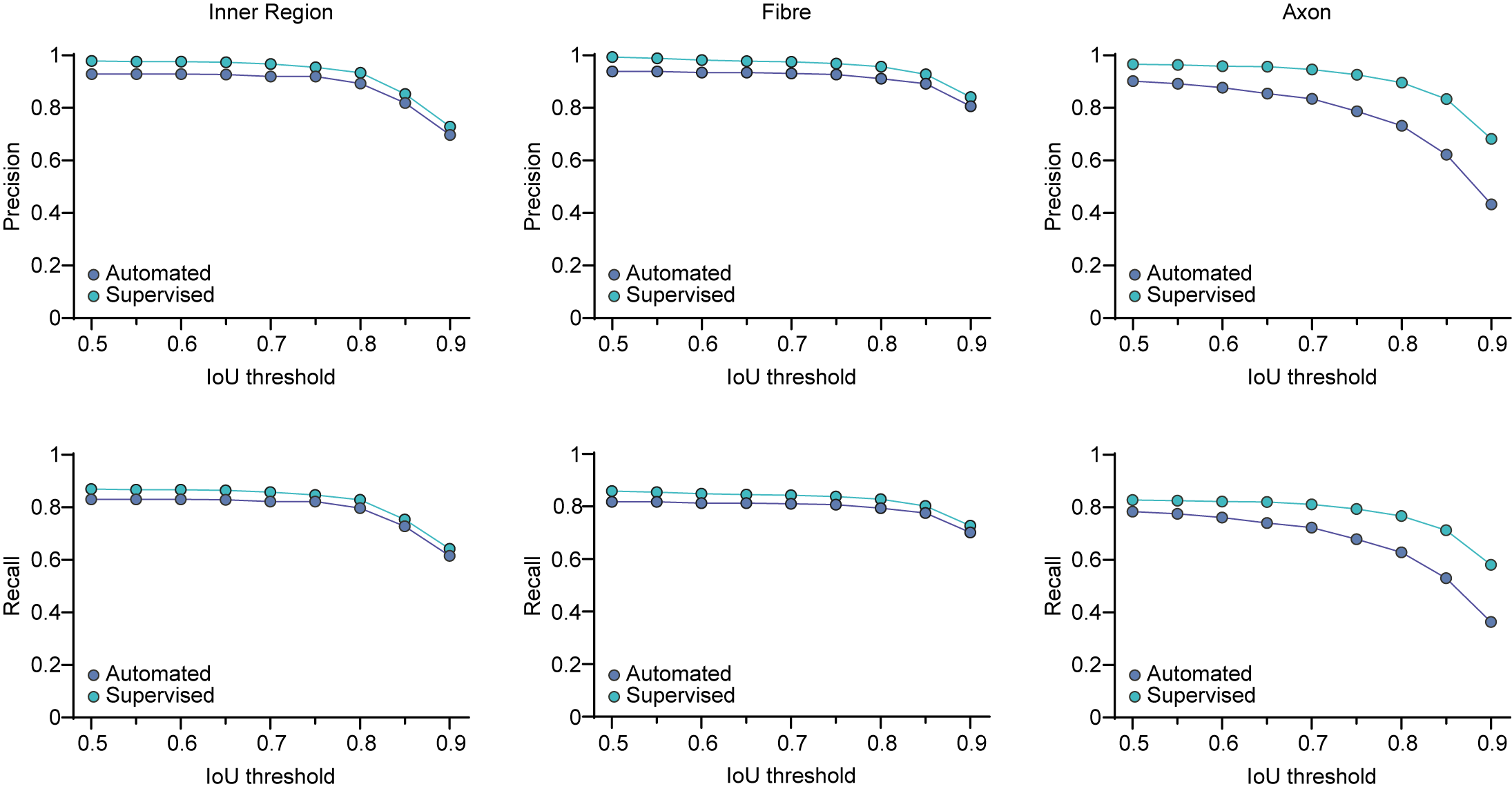

Supplement: S2 Fig — AimSeg was run in automated and supervised modes to compute precision and recall for the segmentation of (A) the inner region, (B) the fibre, and (C) the axon. (TIF) [file pcbi.1010845.s002.tif]

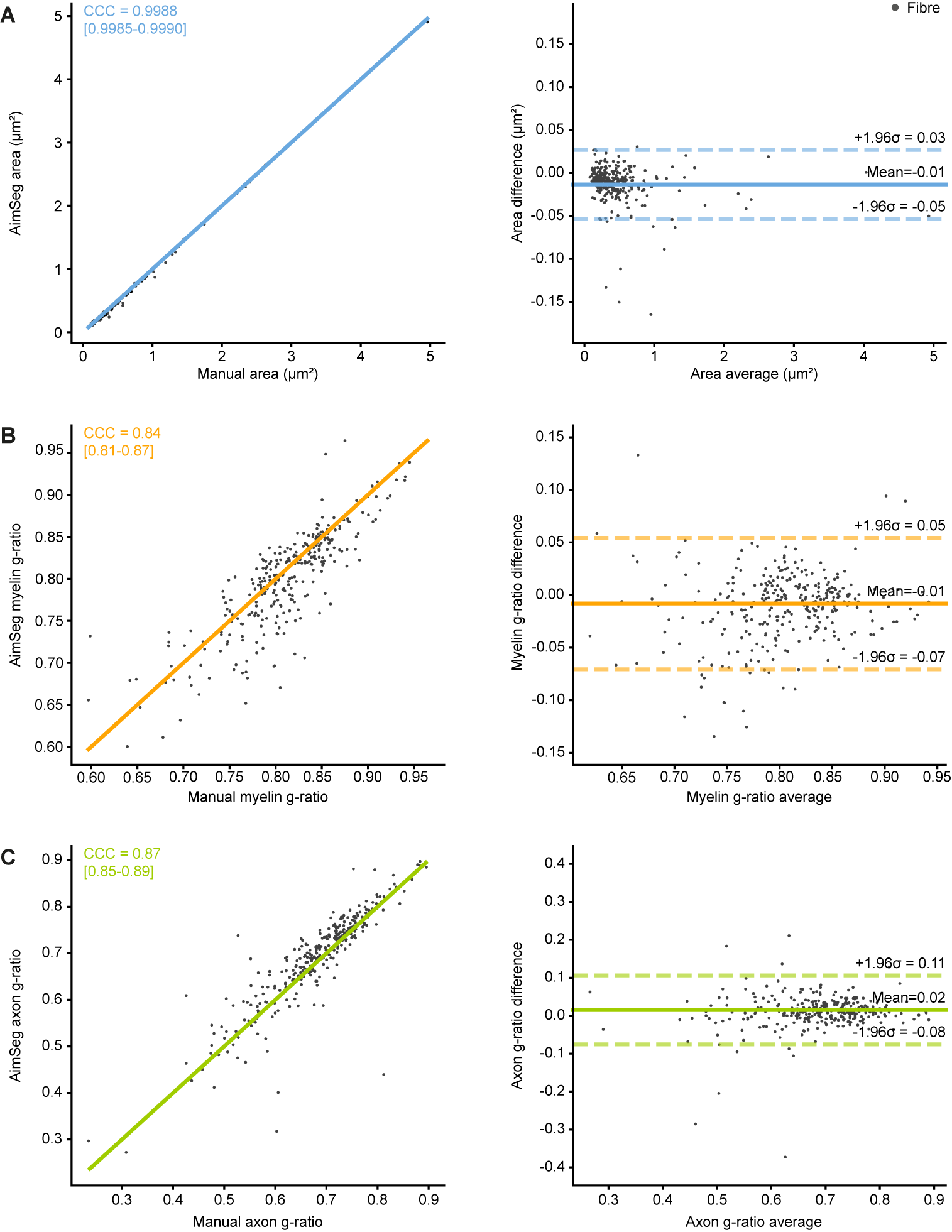

Supplement: S3 Fig — (A) Comparison of the fibre areas obtained by manually segmenting the images or using AimSeg. (A, left) The measurement agreement is calculated as the Lin’s concordance correlation coefficient (CCC), (A, right) while the measurement bias is assessed by means of a Bland-Altman analysis. Diagonal line in the CCC plot represents perfect agreement (y = x). CCC and Bland-Altman plot for (B) the myelin and (C) the axon g-ratios. (TIF) [file pcbi.1010845.s003.tif]

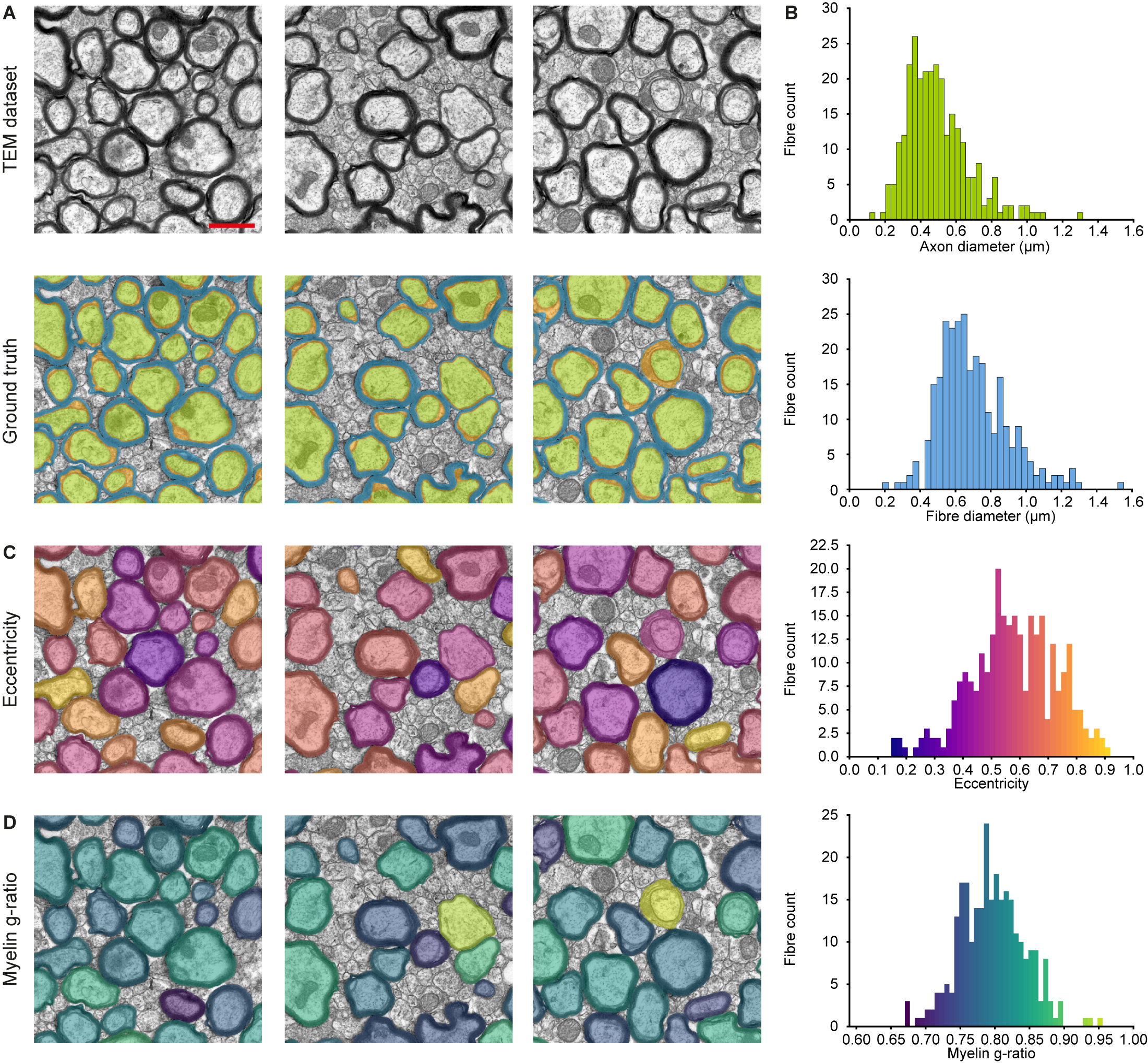

Supplement: S4 Fig — (A, top) Examples of transmission electron microscopy (TEM) images of the corpus callosum from a healthy, adult mouse. Scale bar (red line) = 1 μm. (A, bottom) Manual segmentation of the compacted myelin (blue), the inner tongue, (orange) and the axon (green). (B-D) Diversity of axon/fibre size, shape or myelin thickness. (B) Histograms representing different metrics determined from the manual annotations. (C) The fibres are colour-coded based on the histogram bins to represent the distribution of fibre eccentricity, describing how much a fibre section diverges from a circle, with 0.0 representing a perfect circle. (D) The fibres are colour-coded based on the histogram bins that illustrate their g-ratio distribution (ratio of diameter of the area enclosed by the innermost compact myelin border and the diameter of the whole fibre). Higher g-ratios correspond to thinner myelin, with 1.0 representing the complete absence of myelin sheath. (TIF) [file pcbi.1010845.s004.tif]
